# Addressing disparities in cancer clinical trials: a roadmap to more equitable accrual

**DOI:** 10.3389/frhs.2024.1254294

**Published:** 2024-03-08

**Authors:** Jon A. Hoin, Bradley C. Carthon, Shantoria J. Brown, Lynn M. Durham, L. Crain Garrot, Sharad A. Ghamande, Andrew W. Pippas, Brian M. Rivers, Cindy T. Snyder, Sheryl Gordon Ann Gabram-Mendola

**Affiliations:** ^1^ Rollins School of Public Health, Emory University, Atlanta, GA, United States; ^2^ Department of Hematology and Medical Oncology, Winship Cancer Institute, Emory University, Atlanta, GA, United States; ^3^ Georgia Center for Oncology Research and Education, Atlanta, CO, United States; ^4^ Georgia Cancer Specialists, Atlanta, GA, United States; ^5^ Department of Obstetrics and Gynecology, Augusta University, Augusta, GA, United States; ^6^ Piedmont HealthCare, Columbus, GA, United States; ^7^ Cancer Health Equity Institute, Morehouse School of Medicine, Atlanta, GA, United States; ^8^ Emory University, Atlanta, GA, United States

**Keywords:** cancer care access, clinical trials, disparities, clinical trial accrual, navigation

## Abstract

The Georgia Center for Oncology Research and Education (Georgia CORE) and the Georgia Society of Clinical Oncology (GASCO) held a one-day summit exploring opportunities and evidence-based interventions to address disparities in cancer clinical trials. The purpose of the summit was to identify clear and concise recommendations aimed at decreasing clinical trial accrual disparities in Georgia for rural and minority populations. The summit included expert presentations, panel discussions with leaders from provider organizations throughout Georgia, and breakout sessions to allow participants to critically discuss the information presented. Over 120 participants attended the summit. Recognizing the need for evidence-based interventions to improve clinical trial accrual among rural Georgians and persons of color, summit participants identified four key areas of focus that included: improving clinical trial design, providing navigation for all, enhancing public education and awareness of cancer clinical trials, and identifying potential policy and other opportunities. A comprehensive list of takeaways and action plans was developed in the four key areas of focus with the expectation that implementation of the strategies that emerged from the summit will enhance cancer clinical trial accrual for all Georgians.

## Introduction

In 2022, the Georgia Center for Oncology Research and Education (Georgia CORE) and the Georgia Society of Clinical Oncology (GASCO) held a one-day summit exploring opportunities and evidence-based interventions to address disparities in cancer clinical trials. The summit included expert presentations, panel discussions with leaders from provider organizations throughout Georgia, and breakout sessions to allow participants to critically discuss the information presented.

Participants at the summit sought to build upon recent work by offering clear and concise recommendations aimed at decreasing clinical trial accrual disparities in Georgia. Under-enrollment of minority populations in cancer trials has been an ongoing challenge in cancer research, and it is ultimately detrimental to all people who would benefit from more well-studied cancer treatments (1, 2). Reduced minority participation raises questions about “the generalizability of results for clinical decision making and contributes to persistent racial disparities in cancer outcomes.” (1) Additionally, clinical trials provide access to advanced treatments, and increasing minority enrollment helps address health disparities caused by structural problems (3).

Since patients typically do not choose to enroll at different rates due to skin color, gender or other patient demographics, most disparities in clinical trial accrual are structural in nature, and prospective patients are too often not given opportunities to access cutting edge medicine due to trial availability or geography (3–5). Accrual disparities may also arise as unexpected consequences of trial design (3, 4, 6). Finally, bias and social determinants of health play a role in accrual disparities, and intentional efforts may be required to address existing inequalities to facilitate greater access for patients locked out of trial participation due to circumstances beyond their control (3, 4, 7, 8). By increasing trial availability in rural communities, loosening unnecessary eligibility requirements, and resourcing trial infrastructure, clinical trial access could expand to more than 75% of cancer patients, as opposed to the 5% now participating (3).

Recognizing the need for evidence-based interventions to improve clinical trial accrual among rural Georgians and persons of color, summit participants identified four key areas to focus on:

(a) Improving clinical trial design.
(b) Providing navigation for all.
(c) Enhancing public education and awareness of cancer clinical trials.
(d) Identifying potential policy and other opportunities.

Key stakeholders are encouraged to further develop and enact recommendations in these four areas with the expectation that the set of combined strategies emerging from the summit will enhance cancer clinical trial accrual for Georgians. (Table 1).

**Table 1:** Comprehensive Key Takeaways and Action Plans from Georgia's 2022 Summit on Disparities in Clinical Trial Accrual.

Take away	Action plan
Improving clinical trial design	
Expand minimum eligibility criteria to further increase access to clinical trials: many criteria can be relaxed without risking patient safety.	Convene a panel of experts to assess the 2021 ASCO and Friends of Cancer Research (FCR) recommendations and continue to educate Georgians about new guidelines/recommendations.
Justify minimum eligibility criteria based on scientific criteria, especially where some criteria intersect with social determinants of health; try to accrue representative population samples.	Increase awareness about potential new trials’ eligibility criteria and recognize trials that take steps to ensure their minimum eligibility criteria are expanded yet scientifically justifiable.
Simplify consent forms to enhance patient understanding, and use multi-modal educational tools such as bulleted lists, graphics, plain language, and short sentences.	Inform stakeholders and advocates for consent form simplification. Develop an adaptable Georgia centered educational program and sample materials to teach design principles for simplifying consent forms.
Address cost and geographic barriers by adjusting clinical trial design.	Consider building reimbursements for food, lodging, lost work time, and childcare to remove cost barriers to enroll on a clinical trial. Manage geographic barriers by minimizing patient visits, utilizing telemedicine, opening trials at smaller centers, seeking out deliberately diverse geographic regions, building treatment teams representative of regional diversity, and educating healthcare providers about implicit bias.
Use clinical trial designs to educate about and access somatic and germline testing, giving patients access to essential tools for fighting cancer.	Evaluate strategic opportunities to make somatic and germline testing a focal point of ongoing patient advocacy to promote use in new clinical trials.
Open more clinical trials to focus on the use of precision medicine and this may provide opportunities for patients who may not otherwise have access to a trial.	Support opening more precision medicine trials in Georgia through advocacy, development, and education.
Providing navigation for all	
Design navigation programs that are patient focused and, flexible with the goal of overcoming disparities in clinical trial accrual.	Assess Georgia's clinical trial sites to identify the extent of investment in patient navigation. Design and implement an adaptable professional and patient navigation program for small clinics in rural Georgia. Advocate for universal patient navigation in all clinical trials present in Georgia. Support educational institutions in patient navigation programs/degrees to provide a pipeline of individuals to fulfill these roles.
Employ a spectrum of navigators that include professional nurse or social work navigators or patient navigators to help bridge cultural divides and provide tailored support.	
Enhancing public education and awareness of cancer clinical trials and treatment opportunities	
Focus on access to care, dispelling myths, and offering opportunities for action.	Develop a sample curriculum with simple action items, like handing out conversation cards, to provide to local communities.
Develop peer-to-peer information streams and targeted interventions based on community needs given they are more effective than one-size-fits all approaches.	Recruit and empower local community leaders to become advocates for clinical trial accrual.
Use multimedia tools such as video, animations and decision aids that are inexpensive and easy to maintain.	Design and develop multimedia educational tools for use in Georgia.
Emphasize grass roots relationships and social media outreach tools that are aligned and look like the community. Provide opportunity for a prospective patient to see themselves as potential clinical trial participant.	Launch a social media advocacy campaign aimed at representing the diversity of people in clinical trials. Attach the campaign to specific advocacy events in local communities.
Raise awareness and offer intra-institutional professional education to inform care teams, support personnel and administration about the value of clinical trials as well as implicit bias.	Encourage implicit bias training at Georgia medical institutions and provide continuing education opportunities addressing bias. Encourage principal investigators and their teams to set aside time to educate other institutional personnel on the role and value of clinical trials.
Implement universal screening, informing, and asking all patients about clinical trial participation. This may counter individual implicit biases.	Assess Georgia clinical trial sites, and advocate for implementing universal screening and “just ask” policies where necessary.
Update and inform providers, including those outside oncology such as primary care professionals about the benefits of clinical trials.	Provide extra opportunities for continuing education to providers in Georgia including outreach to primary care providers about how they can appropriately inform patients about clinical trials.
Support healthcare providers with infrastructure to provide both standard of care and access to clinical trials.	Support education to healthcare providers in research programs about methods to increase funding for additional research personnel, patient navigation, and infrastructure.
Identifying potential policy and other opportunities within the state of Georgia and nationally	
Identify potential partners for new technology to decrease the barriers to clinical trial accrual. Blue Button is an example of a powerful new search tool designed to find all clinical trials a patient may qualify for in each geographical distance.	Support the American Cancer Society's interest in recruiting 1–3 clinical implementation partners to test the tool.
Focus on tangible incentives when suggesting new initiatives to policy makers.	Support ASCO as it promotes two federal initiatives which address overall costs and barriers to cancer care.
Boost support for clinical trials that will ultimately benefit all Georgians and the Georgia state economy.	Explore potential programs and advocacy efforts to strengthen state statues related to clinical trials.

### Cancer care in Georgia

Georgia's modern cancer care landscape began to take shape in 2001 with a new initiative aimed at using tobacco settlement funds to move the state from the 4th quartile nationally to the 1st (9, 10). The initiative coordinated public and private investors with academic and community healthcare institutions: regional cancer coalitions formed addressing frontline opportunities such as screening and prevention. The 2001 initiative also launched the state's first Comprehensive Cancer Control Plan which would later be revised to create a “living” document allowing for continuous adjustment as contextual elements change (10).

Progress has been made; however, Georgia still has work to do to become a national leader in both cancer treatment and research (11). An estimated 63,170 Georgians will be diagnosed with cancer in 2024, and 18,740 Georgians will die from cancer this year (12). Georgia has an elevated incidence of cancer, with an age adjusted incidence rate of 463.8 per 100,000 per year, compared with the national average, 442.3 per 100,000 per year (13). Data on clinical trial accrual disparities at the state level can be hard to come by, but trial accrual disparities are well documented at the national level (3, 14, 15).

There is little evidence to suggest that Georgia differs markedly from the national pattern, and if it does, evidence of other treatment disparities imply that trial accrual disparities would exceed national averages. In Georgia, age adjusted incidence rates for black patients are 462.3 per 100,000 per year with white patients at 485.1 per 100,000 per year; however, a look at age adjusted mortality rates reveals an inverse relationship with Black patients dying at a rate of 166.6 per 100,000 per year as opposed to 155.1 for white patients (13). Notably, disparities in overall mortality rates have narrowed over the past 20 years, but they have not disappeared, nor do they reflect the relative incidence of cancer for their respective populations. Black men have a higher lifetime probability of developing and dying from prostate cancer, averaging 40.5 age adjusted deaths in Georgia per 100,000 per year as opposed to white men whose rates are 16.6 deaths per 100,000 per year in Georgia (13).

Furthermore, rural populations in Georgia bear the brunt of the state's cancer burden. 71.1% of Georgia's population, living in 149 of 159 counties, are medically underserved according to state defined criteria, and nearly 54% (85/159) of Georgia's counties are classified as rural based on the 2013 Rural-Urban Continuum Codes (16, 17). Georgia's cancer mortality hotspots are concentrated in the eastern Piedmont to Coastal Plain, southwestern rural Georgia, and northern rural Georgia (16). Hotspot counties generally have a higher proportion of non-Hispanic black adults, older adults, greater poverty, limited access to healthy food, and more rurality (16). For all cancers, age adjusted mortality rates were higher in hotspot counties (16). Differences in outcomes ascribed to rurality are likely related to healthcare access, and when clinical trial data was used to evaluate patient outcomes, rural and urban patients faired similarly, indicating that access to uniform treatment strategies can resolve geographically related disparities in cancer outcomes (6).

## Potential solutions to overcome existing accrual disparities

### Improving clinical trial design

Several promising action items emerged from summit discussions to improve trial design: expanding minimum eligibility criteria, expanding access to precision medicine, and using trial design to proactively address systemic barriers to enrollment.

There is a growing consensus that minimum eligibility criteria are frequently too narrow. A recent series of reports by the American Society of Clinical Oncology (ASCO) and the Friends of Cancer Research (FCR) reviewed common minimum eligibility criteria, finding many to be unnecessarily restrictive at the cost of significantly reducing eligible populations, accrual rates, and excluding historically marginalized populations (18–24). Many typical criteria particularly exclude black patients from clinical trial participation and are often not medically justifiable (25, 26). With excluded patients typically receiving standard of care treatments, several studies have found that patients’ lack of tolerability of standard of care cannot be used to justify excluding them from clinical trials. Patients found to be ineligible, most often because of advanced age and heart disease, go on to tolerate, and even improve, with standard of care treatment, and they are more likely to die from the disease than complications resulting from treatment (27, 28).

Precision medicine provides new opportunities to capture more representative cross sections of patients in the accrual process. Genomic and transcriptomic profiling have proven useful for improving therapy recommendations and patient outcomes, but access to precision medicine, particularly genetic counseling and germline and somatic testing, may be limited by age, ethnicity, and insurance status (29–31). One option for expanding access to genetic counseling and testing is streamlining processes, such as training non-geneticist clinicians to be able to initiate genetic testing, and aiming to implement universal testing while working with existing resources and keeping costs down (29, 32). Expanding access to somatic and germline testing through trial design could be especially useful for addressing barriers related to trial availability. One value of genomic biomarkers is that they may be used in the selection of active immunotherapy or gene directed therapy for patients whose tumor type would not otherwise be individually studied (33). Patients typically ineligible for trials due to availability, if they have a rare or otherwise understudied tumor type for example, may become eligible as trials open that seek cross sections of the population based on genomic biomarkers rather than tumor type.

Finally, trials can be designed to explicitly address common systemic barriers to participation. Major barriers include location and cost. Costs not traditionally covered by insurance, including food, lodging, lost work time, and childcare, may impose significant hurdles to trial participation to families with limited incomes; however, reimbursement opportunities combined with navigation can improve clinical trial accrual (34, 35). Geography also has a major impact on patient costs as it is correlated with travel time, cost, and cultural difference. Overcoming geographic barriers may include efforts to minimize the number of patient visits, open trials at smaller centers, use more telemedicine, seek out geographic diversity, and diversify teams to match the demographics of a given region (36, 37).

### Providing navigation for all

Clinical trial navigation rates extremely high as the most important tool for trial accrual and retention. Several studies have found both professional and lay navigators to be an indispensable resource for educating prospective patients, boosting patient satisfaction, and accruing and maintaining patient participation (3, 38–43). Cancer is one of the most disorienting experiences a person can experience, and navigation helps orient patients toward effective treatments. Professional navigators are particularly helpful in assisting patients with overcoming barriers to participation by employing specialized knowledge to identify open trial opportunities, arranging communication, referrals, service arrangements, and proactive education (42). Lay, also referred to as nonclinical, navigators help to bridge gaps in education, cultural experience, and even language (38–41, 44). Expanded navigation has even proven useful in addressing cancer screening disparities in the state of Delaware, and there is every reason to believe it is an indispensable tool for addressing clinical trial accrual disparities (45, 46).

### Enhancing public education and awareness of cancer clinical trials

One theme that emerges when examining solutions to ending disparities in trial accrual is that patients’ prior education affects their ability to advocate for themselves and make optimal decisions. Likewise, providers make decisions about which patients to try to accrue for clinical trials, and bias, conscious or unconscious, can affect decision making. As such, a robust general education program on clinical trials and implicit bias training for providers may be valuable tools for addressing disparities in clinical trial accrual. Additionally, messaging and education should focus on motivating providers to advocate for clinical trials. By simply educating every patient and their families, screening them, and asking them to participate in open trials, biases may be circumvented.

### Identifying potential policy and other opportunities

Policy at all levels may be used to address areas where education gaps, as well as explicit and implicit bias, may affect clinical trial accrual. Advocates for reducing clinical trial disparities should understand that race is a factor that principal investigators, healthcare providers, and staff may not explicitly consider when accruing patients (47). Choosing not to consider race may fit a practice of moving away from historic patterns of systematized discrimination based on race in the United States, but it comes at the risk of failing to recognize the legacy of barriers implemented with that same systematized discrimination. St. Jude Children's Hospital is a particularly helpful example of one opportunity to combat bias through policy since pediatric oncology, a historically clinical trial centric field, offers almost all patients opportunities to participate in clinical trials. St. Jude's staff, despite a lack of exposure to implicit bias training and a measured preference for high socioeconomic status white individuals, did not differ in recommending patients for trial participation (7). They defaulted to the cultural norms of the field by simply asking everyone. Knowing that acceptance rates do not differ all that much, just asking every single patient could impact accrual disparities (3).

### Barriers to implementing proposed solutions in Georgia and elsewhere

As with many complex problems, improving clinical trial accrual requires addressing multiple factors at different levels of existing healthcare infrastructure. Some solutions are beyond the reach of Georgia, and they will require the collaboration of federal agencies. For example, ordinary Georgians cannot control whether the Centers for Medicare and Medicaid Services (CMS) will pay for patient navigation services, services that may help those seeking a clinical trial as a part of their cancer treatment. CMS is to be applauded for recent efforts to add patient navigation to billable services in the physician fee schedule (48). Further such efforts are needed, but progress is being made.

Many factors are within the control of Georgia's residents, and collaborative efforts can begin to address barriers that exist at the state and local level. Until 2023, the law in Georgia lacked clarity about whether reimbursements for clinical trial patients’ expenses could be considered undue inducements for participation in trials (49, 50). At the time of enactment, Georgia was only the seventh state to codify in law that covering trial participation expenses is legal (49). Legal ambiguity can have a negative effect on innovation designed to address the barriers many people must overcome to participate in clinical trials. Likewise, recent guidance by the FDA acknowledges the human factor in improving clinical trial accrual. They note that many components of eligibility criteria are used as templates across trials without strong clinical or scientific justification (51). Templates are useful because they may save unnecessary labor or guide work. Unfortunately, disrupting the status quo can be challenging and it is often easier to maintain certain processes, ignoring critical reflection on the evidence to support changes, such as eligibility criteria.

Stakeholders seeking to improve clinical trial accrual must keep the human factor in mind. It is not enough to just teach people about the need for equitable accrual. Managers and public health authorities must be able to provide the resources for transition to a better system. People respond well to tools and systems that are user friendly. That means investments that will pay off are updates to existing infrastructure and tools, such as default templates that everyone uses, that smooth the experience of patients and researchers by enabling efficient usage of resources. New incentives may be helpful such as expanded funding tied to equitable accrual requirements. Administrators must also evaluate procedures and processes at the local level. Each organization will need self-examination and proactive planning if this effort is to succeed. Otherwise, organizational inertia will slow or prevent improvements in clinical trial accrual.

## Conclusion

Reducing disparities in clinical trial accrual is a relatively young field of study, and the evidence-based literature for interventions is limited. Georgians are committed to expanding the opportunities to intervene to provide increased access to critical and lifesaving cancer care. One major lesson that emerged from Georgia's summit is that everyone can play a part ranging from advocacy to policy making, and to direct trial design. The potential for significant progress, particularly through changes to trial design alone, is cause for hope. The progress that has been made is worth celebrating as the risk of dying from cancer continues to drop at an accelerated pace and public funding mechanisms are aligned to help those who struggle the most with the cost of cancer (52, 53). Georgia is ready to continue the fight.

## Data Availability

The original contributions presented in the study are included in the article/Supplementary Material, further inquiries can be directed to the corresponding author.
